# The Role of Pharmacotherapy for Excessive Daytime Sleepiness in OSA: A Changing Landscape?

**DOI:** 10.1016/j.chpulm.2026.100245

**Published:** 2026-02-27

**Authors:** Julia L. Chapman, Jian Eu Tai, Claudia Harper, Ronald R. Grunstein, Brendon J. Yee

**Affiliations:** a Sleep and Circadian Research Group, Woolcock Institute of Medical Research, Macquarie University, Macquarie Park, NSW, Australia; b Macquarie Medical School, Faculty of Medicine, Health and Human Sciences, Macquarie University, Sydney, NSW, Australia; c Royal Prince Alfred Hospital, Sydney, NSW, Australia; d Central Clinical School, University of Sydney, Camperdown, NSW, Australia

**Keywords:** excessive daytime sleepiness, incretins, orexin agonists, OSA, pharmacotherapy of sleep apnea, wakefulness promoters

## Abstract

**Topic Importance:**

Excessive daytime sleepiness is a key presenting feature of patients with OSA. When sleepiness remains despite traditional therapies like CPAP, wakefulness-promoting medication may be appropriate. Treating daytime sleepiness directly also may benefit patients who are unable to tolerate CPAP or other mechanical therapies. The growing number of patients with obesity-related OSA may experience sleepiness caused by both OSA and obesity, and weight loss pharmacotherapy may reduce daytime sleepiness.

**Review Findings:**

Several wakefulness promoters are available globally for the indication of residual excessive daytime sleepiness (EDS) in OSA despite CPAP, with some differences in drug access among countries. Recent network meta-analyses have examined the comparative effectiveness of approaches to treat residual EDS in OSA, but clinical guidelines have not been updated since 2013. This review explores the latest evidence on currently available wakefulness promoters and their comparative effectiveness in EDS despite treatment. Evidence for treating sleepiness in the lesser-studied group of patients who are unable to tolerate mechanical therapies for OSA is presented, and the future directions of 2 emerging drug classes are explored, including orexin-2 receptor agonists, with their direct wake-promoting effect, and incretin-based antiobesity agents used in OSA.

**Summary:**

We found that existing medications reduce daytime sleepiness in both patients with EDS despite treatment and in patients with OSA who are not using mechanical therapy, but their effectiveness is limited. Further research is required to determine if newly developed orexin-2 receptor agonists and incretin-based obesity medications could change the clinical landscape to treat sleepiness in OSA.

Excessive daytime sleepiness is a key symptom of OSA and often is the impetus for patients to seek treatment at sleep clinics.^1^ Treatment with traditional mechanical therapies like CPAP can eliminate sleepiness for many, but a large number of patients continue to experience sleepiness. This residual excessive daytime sleepiness (EDS) may be treated with a number of wakefulness-promoting agents that have become available in many countries in recent years.^1^^,^^2^ The US clinical practice guidelines for the medical treatment of sleepiness in OSA have not been updated since 2006^3^ and 2013,^4^ but recent network meta-analyses have attempted to compare efficacy among available agents. In reality, most have small to moderate effects in reducing daytime sleepiness.^5^, ^6^, ^7^, ^8^ Newly discovered orexin-2 receptor agonists have shown early promise in the treatment of excessive sleepiness across central disorders of hypersomnolence and in OSA.^9^, ^10^, ^11^, ^12^, ^13^

Many patients who decline or are unable to tolerate mechanical therapies for OSA still experience sleepiness that can impact their activities of daily living.^4^ These patients represent a conundrum for clinicians, who have limited options to treat them. Wakefulness promoters also may have a role to treat sleepiness in these patients when other treatment options have been exhausted.

For patients with obesity-related OSA, both those tolerant and intolerant of CPAP, new weight-loss pharmacotherapies represent a new frontier in the treatment of the disease.^14^ Obesity itself is associated with daytime sleepiness, independent of OSA.^15^, ^16^, ^17^ Therefore, it is possible that weight reduction in obesity-related OSA may be doubly beneficial in reducing daytime sleepiness.

This review explores the latest evidence for the role of pharmacotherapy to treat EDS in OSA, focusing first on the large number of patients with EDS despite treatment, assessing the comparative effectiveness of currently available medications. The review also explores the usefulness of directly targeting sleepiness as a symptom when mechanical OSA therapies cannot be tolerated. Finally, the future directions of treating sleepiness in OSA are explored, including new wakefulness-promoting orexin-2 receptor agonists and incretin-based antiobesity medications.

## Literature Search

To identify the current best evidence to compare the effectiveness of existing medications to treat EDS despite other OSA treatment, the authors conducted a systematic search on Ovid Medline on August 29, 2024 (updated May 14, 2025). The search strategy combined terms to identify drug therapies for sleepiness in OSA. The complete search criteria can be found in e-Appendix 1.

The inclusion criteria for the analysis of studies evaluating medical treatment of excessive daytime sleepiness despite other OSA treatment were as follows: for patients, with OSA using mechanical device therapy (CPAP); for intervention, any wakefulness-promoting pharmacotherapy; for comparator, placebo or other pharmacotherapy; for outcome, any validated measure of objective (eg, Maintenance of Wakefulness Test [MWT]) or subjective (eg, Epworth Sleepiness Scale [ESS] questionnaire) daytime sleepiness; for study type, randomized controlled clinical trials. Network meta-analyses of randomized controlled clinical trials also were included to evaluate comparative effectiveness better among different wakefulness-promoting agents.

Articles that included primarily patients with EDS despite other OSA treatment are included in Table 1.^18^, ^19^, ^20^, ^21^, ^22^, ^23^, ^24^, ^25^, ^26^, ^27^, ^28^ Articles that included patients with sleepiness who were not using mechanical therapy for OSA are described in the Results. A second systematic search was performed to find the impact of newer incretin therapies on OSA and sleepiness. The search terms can be found in e-Appendix 1. These articles are described in the Results section.

**Table 1:** Studies Evaluating Pharmacotherapy for Residual Sleepiness Despite Other Treatment for OSA

Study	Drug	RES Criteria	OSA or CPAP Criteria	Design, Duration (Sample Size)	Outcomes	Adverse Effects
Kingshott et al (2001)^18^	Modafinil 400 mg vs placebo	ESS score ≥11, MSLT < 10 min	AHI ≥ 15 events/h, at least 4 wk	Randomized crossover trial, 2 wk (N = 30)	MWT: modafinil, 18.3 (3.9) min; placebo, 16.6 (5.0) min (*P* = .02) ESS: no significant difference	
Pack et al (2001)^19^ and Dinges and Weaver (2003)^27^	Modafinil titrated to 400 mg vs placebo	ESS score ≥ 10	AHI ≥ 15 events/h, at least 2 mo	Randomized controlled trial, 4 wk (N = 157)	MSLT: modafinil, 8.6 mins vs placebo, 7.4 mins ESS: modafinil, 9.6 vs placebo, 12.4	Headache (23%), nervousness (12%), nausea (6%), dizziness (6%), anxiety (6%), and insomnia (5%)
Black and Hirshkowitz (2005)^20^	Modafinil 200 mg and 400 mg and placebo	ESS score ≥ 10	OSAH syndrome, at least 4 wk of CPAP treatment	Randomized controlled trial, 12 wk (N = 305)	20-min MWT: modafinil 400 mg, 15 min; 200 mg, 14.8 min; placebo, 12.6 min (*P* < .0001) ESS: modafinil 400 mg, –4.5; 200 mg, –4.5; placebo, –1.8 FOSQ responders: modafinil, 45% vs placebo, 25% (*P* < .01)^a^	Headache (23%-26%), anxiety (6%-8%), insomnia (4%-7%), and hypertension (4%-8%)
Hirshkowitz et al (2006)^21^	Armodafinil 150 mg/d vs placebo	ESS score ≥ 10, CGI-C ≥ 4	OSAH syndrome, at least 4 wk of CPAP treatment	Randomized controlled trial, 12 wk (N = 259)	Mean change in 30-min MWT: armodafinil 150 mg, +2.3 min; placebo, –1.3 min (*P* = .0003) CGI-C improved: armodafinil, 71%; placebo, 53% Armodafinil improved episodic secondary memory (*P* = .0102), fatigue (*P* < .05), ESS score (armodafinil, –5; placebo, –3)	Headache (15%), nausea (5%), diarrhea (5%), and anxiety (5%)
Roth et al (2006)^22^	Armodafinil 150 mg vs armodafinil 250 mg vs placebo	ESS score ≥ 10	OSAH syndrome, at least 4 wk of CPAP treatment	Randomized controlled trial, 12 wk (N = 391)	Mean change in 30-min MWT: armodafinil 150 mg, +1.9 min; placebo, –1.7 min (*P* = .0003) CGI-C: armodafinil, 72%; placebo, 37% No significant differences in outcomes between armodafinil 250 mg and 150 mg daily	Headache (17.6%), nausea (6.1%), diarrhea (4.2%), anxiety (5.3%), and insomnia (6.5%)
Pepin et al (2021)^23^	Pitolisant 20 mg vs placebo	ESS score ≥ 12	OSAH syndrome, at least 3 mo of CPAP treatment	Randomized controlled trial, 12 wk (N = 244)	Mean change in ESS score: pitolisant, –5.5; placebo, –2.8 (*P* < .001) OSLER: no different	Headache (14.8%), and insomnia (9.3%)
Dauvilliers et al (2024)^24^	Pitolisant titrated up to 40 mg vs placebo	ESS score ≥ 12	AHI ≥ 15 events/h^b^	Randomized controlled trial, 12 wk (N = 361)	Mean change in ESS score: 2.6 more with pitolisant vs placebo (95% CI, 1.8-3.4) OSLER fatigue scale: clinical global impressions all improved, trail-making task no different	Headache (6%), insomnia (5%), anxiety (2%), and nightmare (2%)
Schweitzer et al (TONES-3 2019)^25^	Solriamfetol 37.5 mg, 75 mg, 150 mg, and 300 mg vs placebo	ESS score ≥ 10, 40-min MWT < 30 min	OSAH syndrome, previous MAS or surgery, CPAP	Randomized controlled trial, 12 wk (N = 459)	Mean changes in MWT results: solriamfetol 37.5 mg, 4.7 mins; solriamfetol 75 mg, 9.1 mins; solriamfetol 150 mg, 11 mins; solriamfetol 300 mg, 13 mins; placebo, 0.2 mins Mean change in ESS score: solriamfetol 37.5 mg, –5; solriamfetol 75 mg, –5.1; solriamfetol 150 mg, –7.7; solriamfetol 300 mg, 7.9; placebo, –3.3	Headache (10.1%), nausea (7.9%), and reduced appetite (7.6%)
Strollo et al (TONES-4; 2019)^28^	Solriamfetol, clinician titrated vs placebo	ESS score ≥ 10, 40-min MWT < 30 min	OSAH syndrome, previous MAS or surgery, CPAP	Randomized placebo withdrawal study, 6 wk (N = 174)	Mean difference of MWT results: treatment, +11.2 min; placebo, –4.6 min	Headache (9.8%), dry mouth (6.9%), nausea (6.9%), and dizziness (5.7%)
Van Dongen et al (SHARP; 2025)^26^	Solriamfetol, titrated to 150 mg	ESS score > 10	OSAH syndrome, either CPAP ≥ 5 h for ≥ 1 mo or no CPAP for ≥ 1 mo	Randomized crossover trial, 2 wk (N = 59)	Mean difference between treatment and placebo on digit symbol substitution cognitive task small effect size, d = 0.37 Change from baseline on ESS score was 2.1 points (95% CI, 0.7-3.5) higher with treatment than placebo	Nausea (7%) and anxiety (3%)

AHI = apnea-hypopnea index; CGI-C = Clinical Global Impressions of Change; ESS = Epworth Sleepiness Scale; FOSQ = Functional Outcomes of Sleep Questionnaire; MSLT = Multiple Sleep Latency Test; MWT = Maintenance of Wakefulness Test; OSAH = OSA hypopnea (defined as AHI ≥ 15 events/h or AHI ≥ 5 events/h with associated excessive daytime sleepiness [ESS score > 10]); OSLER = Oxford Sleep Resistance Test; RES = residual excessive sleepiness; SHARP = Solriamfetol’s Effect on Cognitive Health in Apnea Participants During a Randomized Placebo-Controlled Study; TONES = Treatment of OSA and Narcolepsy Excessive Sleepiness.

a Secondary analysis (pooled with Pack et al^19^ and Dinges and Weaver^27^).

b Included patients both adherent and nonadherent with CPAP therapy.

## Evidence Review

### Wakefulness-Promoting Agents in Residual Sleepiness Despite CPAP Therapy

The systematic search identified 4 wakefulness-promoting agents tested in randomized controlled trials and now used clinically to treat EDS despite other OSA treatment. Table 1 summarizes the design, characteristics, and findings of the studies.

#### Modafinil

Modafinil is the earliest agent to be studied in the setting of EDS despite other OSA treatment.^18^^,^^19^ Early trials were of short duration (2-4 weeks) and showed statistically significant, but small, effect sizes on both subjective (ESS) and objective (MWT) measures of sleepiness. Measures of cognitive performance and quality of life showed limited effectiveness, likely because of the short duration of treatment and once-daily modafinil administration, which contrasts with the 200-mg bid prescriptions typically used in clinical practice.^18^

Subsequent trials demonstrated a more significant benefit in the use of adjunctive modafinil after 12 weeks of therapy. In 1 trial, 305 individuals with EDS despite other OSA treatment were randomized to receive once-daily modafinil 200 mg, 400 mg, or placebo, reducing ESS score by –4.5 in both the modafinil 200-mg and 400-mg group compared with –1.8 in the placebo group, accompanied by modest improvements in 20-minute MWT results by approximately 2 minutes.^20^

Clinical Global Impression of Change and patient-centered Functional Outcomes of Sleep Questionnaire scores were improved, particularly in the domains of vigilance, productivity, and activity level. Benefits on Functional Outcomes of Sleep Questionnaire scores were found to be independent of baseline subjective sleepiness, suggesting the importance of these wakefulness-promoting medications on daytime function and quality of life, potentially beyond the direct effect on sleepiness.^29^ A secondary analysis of this study found reduced efficacy of modafinil in the 13% of patients who showed suboptimal CPAP use of < 4 h/d, indicating the importance of ongoing and monitored CPAP use to optimize outcomes in patients with EDS that remains after CPAP treatment.^20^^,^^30^ Headache was the most common adverse event (up to 26%), followed by nausea (10%) and insomnia (4%-7%). Hypertension occurred in 4% to 8% of the patients receiving modafinil.

#### Armodafinil

Armodafinil is the R-enantiomer of racemic modafinil with a 10- to 14-hour half-life, achieving higher concentrations later in the day with the putative benefits of sustained attention and wakefulness for longer periods.^31^ In a 12-week randomized clinical trial of 259 patients, MWT results increased by 2.3 minutes with armodafinil vs 1.3 minutes with placebo. Armodafinil also reduced ESS score and fatigue scores and improved overall clinical condition and episodic secondary memory.^21^ Another trial compared armodafinil 150 mg, 250 mg, and placebo,^22^ showing no difference between the doses on MWT latency, Clinical Global Impression of Change, ESS, and fatigue scores compared with placebo. The authors also noted no impact of armodafinil on polysomnographic sleep parameters or CPAP use.^22^ Pooled analyses from the 2 largest armodafinil trials showed improved episodic secondary memory.^31^ The adverse event profile of armodafinil is similar to modafinil, with headache, insomnia, and nausea the most commonly reported events.

#### Pitolisant

Pitolisant is a novel selective histamine H3 antagonist and inverse agonist initially used as a wakefulness-promoting agent in narcolepsy.^23^ In a study of 244 patients with EDS despite other OSA treatment, pitolisant reduced ESS score by 2.6 points more than placebo (95% CI, 1.4-3.9), but no significant difference was found in objective sleep latency measured by the Oxford Sleep Resistance test. To our knowledge, no trial of pitolisant has used the MWT to assess daytime sleepiness. Clinical Global Impressions of Change and fatigue scores were improved significantly. A second trial of pitolisant is harder to interpret because it combined patients with EDS despite using CPAP and patients not using CPAP.^24^ However, an independent patient-level meta-analysis that evaluated just those patients with residual sleepiness after CPAP treatment showed overall a substantial improvement in ESS and Oxford Sleep Resistance sleep latency scores with both 20-mg and 40-mg doses of pitolisant.^32^ They reported that 40-mg doses are superior in patients older than 50 years and when baseline Apnea Hypopnea Index (AHI) is ≥ 15 events/h. The most frequent adverse effects seen with pitolisant are headache (14.8%) and insomnia (9.3%), with no significant differences between doses. No significant changes in BP from baseline were seen.^23^

#### Solriamfetol

Solriamfetol is a dual dopamine and norepinephrine reuptake inhibitor also used as a wakefulness promoter in narcolepsy.^25^ A clinical trial from the Treatment of OSA and Narcolepsy Excessive Sleepiness (TONES) Phase III program, the TONES 3 study, examined the efficacy of solriamfetol compared with placebo, including both clinical subgroups of patients with residual EDS despite other OSA treatment and those not using any therapy for OSA.^25^ Solriamfetol 300 mg reduced MWT sleep latency findings by 13 minutes, in comparison with 0.2 minutes with placebo. The reduction in ESS score at 12 weeks was 7.9 at the maximum dose, compared with 3.3 with placebo. No difference in effect was seen between participants adherent to CPAP and participants not adherent to CPAP.^25^

A randomized withdrawal study of solriamfetol demonstrated effects on MWT and ESS results of similar magnitude over placebo.^28^ It should be noted that 50 participants dropped out of the titration phase, 6 participants because of adverse events and 21 participants (13%) because of not meeting criteria for improvement, suggesting that a substantial subset of nonresponders need to be considered carefully clinically.

The effect of solriamfetol on cognitive tasks also has been assessed showing significant improvements over placebo on the digit symbol substitution task after 2 weeks of therapy.^26^ However, this again was reported in a combined cohort of CPAP users and nonusers, making clinical interpretation difficult.

The most common side effects with solriamfetol are headache (10.1%), nausea (7.9%), and reduced appetite (7.6%). Small increases in systolic BP (2.5 mm Hg; 95% CI, 0.4-4.6), diastolic BP (1.5 mm Hg; 95% CI, 0.3-2.7), and heart rate (2.9 beats/min (95% CI, 1.7-4.1 beats/min) after dose administration compared with placebo at week 12.^24^

### Comparative Efficacy

Although extensive evidence is available on the efficacy of individual wakefulness-promoting agents in improving residual sleepiness despite other OSA treatment, data on the comparative efficacy between them are limited. Direct comparisons from randomized controlled trials between agents are lacking. Four recent network meta-analyses have been published that indirectly compared the effect of different medications on ESS scores, MWT results, and safety.^5^, ^6^, ^7^, ^8^ These meta-analyses provide an overview of the comparative effectiveness of wakefulness promoters in OSA, and a summary of comparative effects on objective and subjective markers of sleepiness is provided in Table 2.

**Table 2:** Comparative Effectiveness of Commercially Available Medications on Objective (MWT) and Subjective (ESS) Sleepiness

Medication	Using CPAP		Not Using CPAP	
	MWT SOL (min)	ESS Score	MWT SOL (mins)	ESS Score
Modafinil	3.1 (1.7-4.7)	2.4 (1.5-3.5)	NR	3.4 (–0.4 to 7.5)
Armodafinil	3.3 (1.6-4.7)	2.0 (1.0-3.1)	NR	0.2 (−1.1 to 1.5)
Solriamfetol	9.5 (7.5-11)	3.3 (2.3-4.2)	2.2 (1.5-3.1)	8.4 (5.3-12)
Pitolisant	NR	2.8 (0.3-5.8)	NR	3.2 (0.1-6.5)

Data are presented as mean difference (95% CI) vs placebo. ESS = Epworth Sleepiness Scale; MWT SOL = Maintenance of Wakefulness Test sleep onset latency; NR = not reported.

Data are modified from Wang et al^7^ and the primary trials included in that meta-analysis and from Chapman et al.^33^

All agents were found to improve subjective sleepiness significantly as measured by the ESS, ranging from 0.9 points to 4.1 points of improvement over placebo.^8^ It is difficult to compare between agents partly because of different ESS score cutoffs in the inclusion criteria ranging from ≥ 10 to ≥ 12, with those with higher baseline ESS scores showing greatest potential improvements.^8^ Solriamfetol has the highest effect on ESS score followed by pitolisant, modafinil, and armodafinil^5^, ^6^, ^7^; however, it seems that overall, when analyses are restricted to just studies of residual sleepiness despite OSA treatment, no agent is superior to others in subjective improvements on ESS.^8^

Comparative effects on improvements in MWT results are difficult to interpret because of a combination of 20-minute, 30-minute, and 40-minute MWT protocols used across trials of different agents. Solriamfetol delayed sleep onset by approximately 10 to 12 minutes over placebo on a 40-minute MWT, which when compared in a network meta-analysis against modafinil and armodafinil, seems to be significantly better.^8^ However, modafinil and armodafinil trials predominantly used 20- to 30-minute MWT protocols, making direct comparison difficult.^34^ The MWT has not been assessed in a trial of pitolisant. Neshat et al^5^ also included and analysis of danavorexton (a novel IV orexin agonist) in their analysis, finding the greatest effect size on MWT. Neshat et al did not differentiate between studies of patients with residual sleepiness despite using CPAP and studies of patients not using CPAP, so the results are difficult to interpret for clinical populations and should be treated with caution.

It does seem that pitolisant and solriamfetol have fewer adverse events and pitolisant fewer cardiovascular side effects compared with modafinil and armodafinil. Modafinil and armodafinil showed the highest rates of treatment discontinuation because of adverse events in the trials, whereas pitolisant showed the lowest rates.^7^^,^^8^ Modafinil and solriamfetol were found to have a greater cardiovascular risk profile than pitolisant and placebo.^6^ Taking this into consideration, on a benefit to risk analysis, the medications ranked from most to least favorable are pitolisant, solriamfetol, and modafinil/armodafinil. In Europe, modafinil and armodafinil are not indicated for the treatment of OSA, and pitolisant and solriamfetol are used preferentially for patients with cardiovascular risk and obesity, respectively.^2^

Several differences between the randomized studies confound indirect comparisons, for example, treatment duration where different effects within and between agents are found when they are used for ≤ 4 or between 4 and 12 weeks.^7^^,^^8^ No randomized controlled trial of residual sleepiness despite OSA treatment for longer than 12 weeks is available, so longer-term impacts in this population cannot be elucidated from these trial reports. It is also possible that the duration of CPAP therapy may have impacted results between trials, because both the Apnea Positive Pressure Long-term Efficacy Study (APPLES) and European Sleep Apnea Database (ESADA) studies previously demonstrated continued improvement in ESS score with CPAP up to 6 months, which may have affected patients included who recently initiated CPAP therapy.^35^^,^^36^

### Wakefulness-Promoting Agents in Patients With OSA Unable to Use CPAP

Patients with OSA who are unable to tolerate standard treatments that reduce OSA severity like CPAP are a unique subset of patients that may become lost from traditional clinical referral pathways for OSA. It is common for sleepiness to remain in these patients, and treating sleepiness directly with pharmacotherapy may be beneficial for some patients. It is important to note that although wakefulness promoters may be useful to treat the symptom of sleepiness, they do not reduce OSA severity. Modafinil can improve sleepiness during short-term withdrawal from CPAP, suggesting its potential use when a break from CPAP may be wanted or required by patients.^37^ In mild OSA, our research showed that modafinil can improve subjective sleepiness, as measured by ESS score, by 3.2 points over placebo.^38^ Although direct comparisons cannot be made, a meta-analysis of CPAP to reduce sleepiness in mild OSA showed only a 1.2-point reduction over sham CPAP, suggesting that targeting sleepiness directly may be beneficial for some patients with milder OSA.^39^

Additionally, our group has shown that armodafinil can be used adjunctively with weight loss, with greater improvement on 90-minute driving simulator performance at 3 months of armodafinil therapy over placebo.^33^ This result was not sustained to 6 months, with a suggestion that the driving simulator performance may have sustained a learning effect. However, of interest in this trial, armodafinil reduced fat mass by 2.4 kg over placebo and resulted in increased daytime activity as measured by wrist actigraphy, suggesting that it may impart effects both directly on sleepiness and secondarily through weight loss.

Pitolisant also has been studied in this population, significantly reducing ESS score compared with placebo (–6.3 vs –3.6; *P* < .001), but not objective sleepiness on the Oxford Sleep Resistance assessment. No effects on BP or cardiovascular events were seen in this 12-week study.^40^

The TONES-3 study of solriamfetol in OSA described already and in Table 1 included both patients using CPAP and those who had rejected CPAP, showing improvements in both subjective and objective measures of sleepiness.^25^ These findings were replicated in the more recent Solriamfetol’s Effect on Cognitive Health in Apnea Participants During a Randomized Placebo-Controlled Study (SHARP) trial, which showed improvements in cognitive task performance as well as reductions in sleepiness, regardless of CPAP use in patients with OSA taking solriamfetol.^26^ Although the clinical interpretation of this mixed group can be difficult, it seems that solriamfetol can benefit patients not using CPAP to a similar degree as those actively using CPAP.

## Future Directions

### Orexin Agonists

Orexin neurons are found in the lateral hypothalamus and play a key role in sleep-wake regulation. Orexin deficiency is implicated in narcolepsy with cataplexy.^41^ Newly developed orexin-2 receptor agonists are still being trialed, but have shown great potential in improving daytime sleepiness, not just in narcolepsy type 1,^10^^,^^12^^,^^33^^,^^42^ but in other disorders of hypersomnolence, where orexin levels are thought to be normal (eg, narcolepsy type 2, idiopathic hypersomnia).^9^ In animal models, intermittent hypoxia from sleep apnea has been shown to cause injury to orexin neurons.^43^^,^^44^ This is the basis for a small phase 1 study that examined the effect of danavorexton, an orexin-2 receptor agonist, in patients with residual sleepiness despite other OSA treatment. In this proof-of-concept trial, participants were given a single IV infusion of placebo or danavorexton doses of 44 mg or 112 mg. The mean sleep latencies on a 40-minute MWT were 12.4 minutes, 35.3 minutes, and 39.8 minutes, respectively, demonstrating a dose-effect response^11^ that surpasses the improvements seen with existing agents (Table 2). Nevertheless, this was an early phase evaluation of a so-called IV prototype, and it is too early for clinical comparisons to be made. Further testing of emerging oral orexin receptor agonists is needed to determine the potential for these agents to treat sleepiness in OSA.

It will be important for these upcoming agents to differentiate their ability to improve daytime sleepiness, but importantly their safety, tolerability, and ability to improve tests of real-world daytime performance.^45^ A key determinant of the viability of these medications is the risk to benefit profile, particularly in disorders where orexin levels are normal. In narcolepsy type 1, where orexin levels are depleted, lower orexin agonist doses are required to have a marked wakefulness-promoting effect. In other disorders of excessive sleepiness, it seems that higher orexin agonist doses will be required to achieve the same wakefulness effects.^13^ The main side effects are insomnia and increased urinary frequency.^12^^,^^46^ An early orexin agonist clinical trial was ceased because of liver toxicity^10^; however, this was found to be the result of a toxic metabolite, and not a direct drug effect (and therefore not expected to occur with newer agents).

### The Role of Obesity and Obesity Reduction in Daytime Sleepiness

Being overweight and having obesity are the primary modifiable risk factors for OSA, and the 2 conditions often coexist. Although OSA is likely to be a key cause of sleepiness in patients with both obesity and OSA, where sleepiness continues despite CPAP use, obesity could be considered as a potential causative factor of residual sleepiness. Evidence is available that shows that obesity, independent of OSA, is associated with increased daytime sleepiness.^15^, ^16^, ^17^^,^^47^^,^^48^ The mechanisms of this are complex, with a suggestion that feeding behavior may contribute to sleepiness,^49^ because saturated fat intake has been associated with increased propensity for sleepiness.^50^ Additionally, several mechanisms unrelated to OSA have been proposed, including heightened sympathetic activity, disruptions in metabolic and inflammatory pathways, hormonal imbalances, and comorbid obesity-related conditions such as diabetes and depression, all of which can impair nighttime sleep.^17^ Sleepiness itself, even in the absence of any sleep disorder, has been postulated as a potential independent marker of cardiovascular risk, with the complex interactions among obesity, feeding behavior, the gut microbiome, and systemic inflammation all implicated in this relationship.^51^

Evidence is available to suggest that reducing weight can improve daytime sleepiness in patients with OSA who are overweight, but that CPAP did not further enhance this effect, suggesting that weight reduction is having an effect independent of the effect of CPAP.^52^ In individuals with or without OSA, intentional weight loss demonstrates a dose-response relationship between change in weight or BMI and reduction in daytime sleepiness independent of a change in OSA severity (measured by AHI) for those with OSA. Notably, reductions in AHI among those with OSA did not show a corresponding dose-response relationship with reduced sleepiness.^48^ It is possible that daytime sleepiness could be improved with weight loss, regardless of whether OSA or AHI can be improved. As incretin-based pharmacotherapies emerge as first-line therapy for obesity-related OSA,^53^ it is possible that these medications could have a double effect on reducing sleepiness via their reduction of OSA and the reduction in obesity itself. Looking outside of OSA, small reductions in sleepiness have been seen in patients with obesity and diabetes taking liraglutide^54^ and exenatide,^55^ further suggesting that obesity reduction itself may reduce sleepiness directly. A recent meta-analysis reports on the effects of incretin therapies in OSA, showing improvements in AHI, weight reduction, and BP compared with placebo, yet no report of sleepiness was available.^56^ Sleepiness was reported directly in only 1 of the included clinical trials of liraglutide,^57^ and although overall no difference in sleepiness over placebo was reported, supplementary analyses showed that reduction in daytime sleepiness correlated with greater weight loss, suggesting that more substantial weight loss may lead to greater improvements in sleepiness. This is shown in patients with OSA and obesity after bariatric surgery, when substantial weight loss can lead to rapid and enduring improvements in sleepiness, potentially independent of the AHI-reducing effects of that weight loss.^17^ Subjective sleepiness measured via the ESS did not improve on tirzepatide over placebo in patients usin CPAP (mean score, 0.9; 95% CI, –0.2 to 2.1), yet for patients not using CPAP, tirzepatide led to a small but significant improvement in ESS score over placebo (mean, 1.4; 95% CI, 0.3-2.5; *P* < .05).^58^ Other markers of daytime functioning including the Patient-Reported Outcomes Measurement Information System (PROMIS) Sleep-Related Impairment questionnaire, which asks questions about difficulty with daily activities resulting from sleepiness, was improved significantly with tirzepatide over placebo after 52 weeks of treatment (difference in PROMIS Sleep-Related Impairment standardized T score, –3.9; 95% CI, –5.7 to –2.2; *P* < .001).^53^

Studying sleepiness in these trials can be challenging methodologically because patients with both OSA and concomitant excessive sleepiness often are excluded for safety reasons,^59^ or they are recruited from endocrinology and bariatric clinics and do not represent the usual sleepy patients seeking treatment at sleep clinics.^58^ The potential to use wakefulness promoters in future trials of weight loss pharmacotherapy should be considered as a method to overcome initial sleepiness while patients lose weight.

The most common adverse events that occur while taking incretin medications are mild to moderate gastrointestinal effects, which usually occur during dose titration and resolve thereafter.^60^ A clear need exists for further study on the connection among obesity, daytime sleepiness, and OSA and how treatment with weight loss can improve important clinical patient-centered outcomes. This requires longer-term large randomized controlled comparative effectiveness trials with key clinical end points of interest including sleepiness.

## Summary

Of the currently available wakefulness promoters, some evidence suggests that pitolisant and solriamfetol have a marginally superior wakefulness-promoting effect over modafinil and armodafinil in OSA, yet data on objective markers of sleepiness in patients not using other treatments for OSA are limited. Further research is needed on an emerging wakefulness promoter drug class, orexin agonists, to determine their effect in OSA. Sleep clinicians should consider the role that obesity may have on the pathogenesis of excessive daytime sleepiness in OSA and that treating obesity may reduce sleepiness and sleepiness-related impairments.

## Funding/Support

The authors have reported to *CHEST Pulmonary* that no funding was received for this study.

## Financial/Nonfinancial Disclosures

The authors have reported to *CHEST Pulmonary* the following: all authors report funding to their department by Alkermes, Takeda, Jazz Pharmaceuticals, Vanda Pharmaceuticals, and Eli Lilly & Company. B. J. Y. has received funding from Alkermes, Eli Lilly & Company, GlaxoSmithKline, SomnoMed, Takeda, Teva Pharmaceuticals, and Vanda Pharmaceuticals. R. R. G. has received funding from Apnimed, Eli Lilly & Company, and SomnoMed.

## Supplementary Data

e-Online Data

## References

[1] Craig S., Pepin J.L., Randerath W. (2022). Investigation and management of residual sleepiness in CPAP-treated patients with obstructive sleep apnoea: the European view. Eur Respir Rev.

[2] Bonsignore M.R., Francesco F., Pietro I. (2025). Management options for excessive daytime sleepiness in patients with obstructive sleep apnea. Expert Rev Respir Med.

[3] Morgenthaler T.I., Kapen S., Lee-Chiong T. (2006). Practice parameters for the medical therapy of obstructive sleep apnea. Sleep.

[4] Strohl K.P., Brown D.B., Collop N. (2013). An official American Thoracic Society Clinical Practice Guideline: sleep apnea, sleepiness, and driving risk in noncommercial drivers. An update of a 1994 statement. Am J Respir Crit Care Med.

[5] Neshat S.S., Heidari A., Henriquez-Beltran M. (2024). Evaluating pharmacological treatments for excessive daytime sleepiness in obstructive sleep apnea: a comprehensive network meta-analysis and systematic review. Sleep Med Rev.

[6] Pepin J.L., Lehert P., Ben Messaoud R. (2024). Comparative efficacy, safety and benefit/risk of alerting agents for excessive daytime sleepiness in patients with obstructive sleep apnoea: a network meta-analysis. EClinicalMedicine.

[7] Wang Y., Zhang W., Ye H., Xiao Y. (2024). Excessive daytime sleepiness in obstructive sleep apnea: indirect treatment comparison of wake-promoting agents in patients adherent/nonadherent to primary OSA therapy. Sleep Med Rev.

[8] Tanayapong P., Tantrakul V., Liamsombut S. (2025). Comparative efficacy and safety of multiple wake-promoting agents for the treatment of residual sleepiness in obstructive sleep apnea despite continuous positive airway pressure: a systematic review and network meta-analysis of randomized controlled trials. CNS Drugs.

[9] Mignot E., Bogan R.K., Emsellem H. (2023). Safety and pharmacodynamics of a single infusion of danavorexton in adults with idiopathic hypersomnia. Sleep.

[10] Dauvilliers Y., Mignot E., Del Rio Villegas R. (2023). Oral orexin receptor 2 agonist in narcolepsy type 1. N Engl J Med.

[11] Bogan R.K., Maynard J.P., Neuwirth R. (2023). Safety and pharmacodynamics of a single infusion of danavorexton in adults with obstructive sleep apnea experiencing excessive daytime sleepiness despite adequate use of CPAP. Sleep Med.

[12] Dauvilliers Y., Plazzi G., Mignot E. (2025). Oveporexton, an oral orexin receptor 2-selective agonist, in narcolepsy type 1. N Engl J Med.

[13] Grunstein R., Yee B., Chapman J. (2025). 0838 the orexin 2 receptor agonist alks 2680 in patients with narcolepsy or idiopathic hypersomnia: a phase 1b study. Sleep.

[14] Grunstein R.R., Wadden T.A., Chapman J.L., Malhotra A., Phillips C.L. (2023). Giving weight to incretin-based pharmacotherapy for obesity-related sleep apnea: a revolution or a pipe dream?. Sleep.

[15] Vgontzas A.N., Bixler E.O., Tan T.L. (1998). Obesity without sleep apnea is associated with daytime sleepiness. Arch Intern Med.

[16] Bixler E.O., Vgontzas A.N., Lin H.M. (2005). Excessive daytime sleepiness in a general population sample: the role of sleep apnea, age, obesity, diabetes, and depression. J Clin Endocrinol Metab.

[17] Panossian L.A., Veasey S.C. (2012). Daytime sleepiness in obesity: mechanisms beyond obstructive sleep apnea—a review. Sleep.

[18] Kingshott R.N., Vennelle M., Coleman E.L. (2001). Randomized, double-blind, placebo-controlled crossover trial of modafinil in the treatment of residual excessive daytime sleepiness in the sleep apnea/hypopnea syndrome. Am J Respir Crit Care Med.

[19] Pack A.I., Black J.E., Schwartz J.R., Mathesonet J.K. (2001). Modafinil as adjunct therapy for daytime sleepiness in obstructive sleep apnea. Am J Respir Crit Care Med.

[20] Black J.E., Hirshkowitz M. (2005). Modafinil for treatment of residual excessive sleepiness in nasal continuous positive airway pressure-treated obstructive sleep apnea/hypopnea syndrome. Sleep.

[21] Hirshkowitz M., Black J.E., Wesnes K. (2007). Adjunct armodafinil improves wakefulness and memory in obstructive sleep apnea/hypopnea syndrome. Respir Med.

[22] Roth T., White D., Schmidt-Nowara W. (2006). Effects of armodafinil in the treatment of residual excessive sleepiness associated with obstructive sleep apnea/hypopnea syndrome: a 12-week, multicenter, double-blind, randomized, placebo-controlled study in nCPAP-adherent adults. Clin Ther.

[23] Pépin J.L., Georgiev O., Tiholov R. (2021). Pitolisant for residual excessive daytime sleepiness in OSA patients adhering to CPAP: a randomized trial. Chest.

[24] Dauvilliers Y., Craig S.E., Bonsignore M.R. (2024). Pitolisant 40 mg for excessive daytime sleepiness in obstructive sleep apnea patients treated or not by CPAP: randomised phase 3 study. J Sleep Res.

[25] Schweitzer P.K., Rosenberg R., Zammit G.K. (2019). Solriamfetol for excessive sleepiness in obstructive sleep apnea (TONES 3). A randomized controlled trial. Am J Respir Crit Care Med.

[26] Van Dongen H.P.A., Leary E.B., Drake C. (2025). Results of the Solriamfetol’s Effect on Cognitive Health in Apnea Participants During a Randomized Placebo-Controlled Study (SHARP): a randomized placebo-controlled double-blind repeated-measures crossover phase IV clinical trial of the effect of the wake-promoting agent solriamfetol on cognitive function in OSA with excessive daytime sleepiness and cognitive impairment. Chest.

[27] Dinges D.F., Weaver T.E. (2003). Effects of modafinil on sustained attention performance and quality of life in OSA patients with residual sleepiness while being treated with nCPAP. Sleep Med.

[28] Strollo P.J., Hedner J., Collop N. (2019). Solriamfetol for the treatment of excessive sleepiness in OSA: a placebo-controlled randomized withdrawal study. Chest.

[29] Weaver T.E., Chasens E.R., Arora S. (2009). Modafinil improves functional outcomes in patients with residual excessive sleepiness associated with CPAP treatment. J Clin Sleep Med.

[30] Chapman J.L., Serinel Y., Marshall N.S., Grunstein R.R. (2016). Residual daytime sleepiness in obstructive sleep apnea after continuous positive airway pressure optimization: causes and management. Sleep Med Clin.

[31] Roth T., Rippon G.A., Arora S. (2008). Armodafinil improves wakefulness and long-term episodic memory in nCPAP-adherent patients with excessive sleepiness associated with obstructive sleep apnea. Sleep Breath.

[32] Testelmans D., Lehert P., Asin J. (2025). Efficacy and safety of pitolisant in residual excessive daytime sleepiness for patients with obstructive sleep apnea adhering to continuous positive airway pressure therapy in the HAROSA studies: an individual patient meta-analytical approach. Sleep Med.

[33] Chapman J.L., Cayanan E.A., Hoyos,et C.M. (2018). Does armodafinil improve driving task performance and weight loss in sleep apnea? A randomized trial. Am J Respir Crit Care Med.

[34] Chapman J.L., Vakulin A., Hedner J., Yee B.J., Marshall N.S. (2016). Modafinil/armodafinil in obstructive sleep apnoea: a systematic review and meta-analysis. Eur Respir J.

[35] Bonsignore M.R., Pepin J.L., Cibella F. (2021). Excessive daytime sleepiness in obstructive sleep apnea patients treated with continuous positive airway pressure: data from the European Sleep Apnea Database. Front Neurol.

[36] Kushida C.A., Nichols D.A., Holmes T.H. (2012). Effects of continuous positive airway pressure on neurocognitive function in obstructive sleep apnea patients: the Apnea Positive Pressure Long-term Efficacy Study (APPLES). Sleep.

[37] Williams S.C., Marshall N.S., Kennerson,et M. (2010). Modafinil effects during acute continuous positive airway pressure withdrawal: a randomized crossover double-blind placebo-controlled trial. Am J Respir Crit Care Med.

[38] Chapman J.L., Kempler L., Chang C.L. (2014). Modafinil improves daytime sleepiness in patients with mild to moderate obstructive sleep apnoea not using standard treatments: a randomised placebo-controlled crossover trial. Thorax.

[39] Marshall N.S., Barnes M., Travier N. (2006). Continuous positive airway pressure reduces daytime sleepiness in mild to moderate obstructive sleep apnoea: a meta-analysis. Thorax.

[40] Dauvilliers Y., Verbraecken J., Partinen M. (2020). Pitolisant for daytime sleepiness in patients with obstructive sleep apnea who refuse continuous positive airway pressure treatment. A randomized trial. Am J Respir Crit Care Med.

[41] Pizza F., Barateau L., Dauvilliers Y., Plazzi G. (2022). The orexin story, sleep and sleep disturbances. J Sleep Res.

[42] Marshall N.S., Grunstein R.R. (2023). Orexin agonists—two steps forward, one step back. N Engl J Med.

[43] Veasey S.C., Davis C.W., Fenik P. (2004). Long-term intermittent hypoxia in mice: protracted hypersomnolence with oxidative injury to sleep-wake brain regions. Sleep.

[44] Li Y., Panossian L.A., Zhang J., t (2014). Effects of chronic sleep fragmentation on wake-active neurons and the hypercapnic arousal response. Sleep.

[45] Chapman J.L., Marshall N.S. (2025). Sleepy heads no more: can we improve cognitive performance with wakefulness promoters?. Chest.

[46] Mignot E., Bogan R., Del Rio,et R. (2024). Efficacy and safety of TAK-861, an oral orexin receptor 2 agonist, in individuals with narcolepsy type 2: results from phase 2 studies. J Sleep Res.

[47] Vgontzas A.N., Papanicolaou D.A., Bixler,et E.O. (2000). Sleep apnea and daytime sleepiness and fatigue: relation to visceral obesity, insulin resistance, and hypercytokinemia. J Clin Endocrinol Metab.

[48] Ng W.L., Stevenson C.E., Wong E. (2017). Does intentional weight loss improve daytime sleepiness? A systematic review and meta-analysis. Obes Rev.

[49] Pickett S.M., Jacques-Tiura A.J., Echeverri-Alvarado B., Sheffler J.L., Naar S. (2022). Daytime sleepiness, addictive-like eating, and obesity sequelae in Black and African American youth with obesity. Sleep Health.

[50] Melaku Y.A., Reynolds A.C., Gill T.K., Appleton S., Adams R. (2019). Association between macronutrient intake and excessive daytime sleepiness: an iso-caloric substitution analysis from the North West Adelaide Health Study. Nutrients.

[51] Bock J., Covassin N., Somers V. (2022). Excessive daytime sleepiness: an emerging marker of cardiovascular risk. Heart.

[52] Hoyos C.M., Machan E.A., Yeeet al B.J. (2024). Cardio-metabolic health effects of CPAP treatment for sleep apnoea during weight loss: a randomised controlled pilot trial. Obes Res Clin Pract.

[53] Malhotra A., Grunstein R.R., Fietze I. (2024). Tirzepatide for the treatment of obstructive sleep apnea and obesity. N Engl J Med.

[54] Gomez-Peralta F., Abreu C., Castro,et J.C. (2015). An association between liraglutide treatment and reduction in excessive daytime sleepiness in obese subjects with type 2 diabetes. BMC Endocr Disord.

[55] Idris I., Abdulla H., Tilbrook S., Dean R., Ali N. (2013). Exenatide improves excessive daytime sleepiness and wakefulness in obese patients with type 2 diabetes without obstructive sleep apnoea. J Sleep Res.

[56] Li M., Lin H., Yang Q. (2024). Glucagon-like peptide-1 receptor agonists for the treatment of obstructive sleep apnea: a meta-analysis. Sleep.

[57] Blackman A., Foster G.D., Zammit G. (2016). Effect of liraglutide 3.0 mg in individuals with obesity and moderate or severe obstructive sleep apnea: the SCALE Sleep Apnea randomized clinical trial. Int J Obes (Lond).

[58] Kanu C., Shinde S., Chakladar S. (2025). Effect of tirzepatide treatment on patient-reported outcomes among SURMOUNT-OSA participants with obstructive sleep apnea and obesity. Sleep Med.

[59] O’Donnell C., Crilly S., O’Mahony A. (2023). Continuous positive airway pressure but not GLP1-mediated weight loss improves early cardiovascular disease in obstructive sleep apnea: a randomized proof-of-concept study. Ann Am Thorac Soc.

[60] Aronne L.J., Horn D.B., Roux C.W.l. (2025). Tirzepatide as compared with semaglutide for the treatment of obesity. N Engl J Med..

