# Supplementary material for: The Role of Pharmacotherapy for Excessive Daytime Sleepiness in OSA: A Changing Landscape?
Source: CHEST Pulm. 2026 Feb 27;4(2):100245. doi: 10.1016/j.chpulm.2026.100245 (PMC13418711; doi:10.1016/j.chpulm.2026.100245)
Supplement: e-Online Data [file mmc1.docx]

**Search strategy for studies of wakefulness promoters and OSA**

Performed August 2024 and Repeated May 2025

Ovid MEDLINE(R) ALL <1946 to May 14, 2025>

1 sleep apnea syndromes/ or sleep apnea, obstructive/ 44988

2 (obstructive sleep apnea* or sleep apnea hypopnea syndrome* or obstructive sleep apnea syndrome* or upper airway resistance sleep apnea syndrome).ti,ab. 34169

3 ((sleep or nocturnal) adj2 (hypopnea* or hypopnoea* or "hypo‐apnoea*" or "hypo‐apnea*" or "apneic‐hypopneic" or "apnoeic‐hypopnoeic")).ti,ab. 3123

4 ((sleep$ or nocturnal) adj2 (apnea* or apnoea*)).ti,ab. 51605

5 (sleep* adj2 disordered adj2 breathing).ti,ab. 8386

6 (OSA or SAHS or OSAHS).ti,ab. 25314

7 1 or 2 or 3 or 4 or 5 or 6 65375

8 Sleepiness/ 1071

9 (sleepiness or somnolence or sleepy or epworth or vigilance).ti,ab. 46466

10 8 or 9 46556

11 Wakefulness-Promoting Agents/ 266

12 Central Nervous System Stimulants/ 22881

13 Histamine H3 Antagonists/ 361

14 Modafinil/ 1505

15 Dopamine Uptake Inhibitors/ 6402

16 (solriamfetol or modafinil or pitolisant or wakefulness-promot* or stimulant).ti,ab. 21722

17 armodafinil.ti,ab. 229

18 Orexins/ 4052

19 Orexin Receptors/ 1740

20 11 or 12 or 13 or 14 or 15 or 16 or 17 or 18 or 19 50330

21 7 and 10 and 20 289

**Search strategy for studies of obesity management, OSA and sleepiness**

Performed Dec 2024 and repeated 14 May 2025

Ovid MEDLINE(R) ALL <1946 to May 14, 2025>

1 sleep apnea syndromes/ or sleep apnea, obstructive/ 44988

2 (obstructive sleep apnea* or sleep apnea hypopnea syndrome* or obstructive sleep apnea syndrome* or upper airway resistance sleep apnea syndrome).ti,ab. 34169

3 ((sleep or nocturnal) adj2 (hypopnea* or hypopnoea* or "hypo‐apnoea*" or "hypo‐apnea*" or "apneic‐hypopneic" or "apnoeic‐hypopnoeic")).ti,ab. 3123

4 ((sleep$ or nocturnal) adj2 (apnea* or apnoea*)).ti,ab. 51605

5 (sleep* adj2 disordered adj2 breathing).ti,ab. 8386

6 (OSA or SAHS or OSAHS).ti,ab. 25314

7 1 or 2 or 3 or 4 or 5 or 6 65375

8 Sleepiness/ 1071

9 (sleepiness or somnolence or sleepy or epworth or vigilance).ti,ab. 46466

10 8 or 9 46556

11 obesity/ or obesity, abdominal/ or obesity, metabolically benign/ or obesity, morbid/ 265363

12 (obes* or overweight or weight loss).ti,ab. 524618

13 11 or 12 570225

14 exp incretins/ or exp exenatide/ or exp gastric inhibitory polypeptide/ or exp glucagon-like peptide 1/ or exp liraglutide/ 19785

15 (GLP-1 or GIP).ti,ab. 19432

16 (glucagon-like peptide or gastric inhibitory).ti,ab. 21920

17 glucagon-like peptide 1/ or liraglutide/ 12694

18 glucagon-like peptide-1 receptor agonists/ or exenatide/ or liraglutide/ or tirzepatide/ 8576

19 (tirzepatide or semaglutide or exanatide or liraglutide).ti,ab. 7113

20 14 or 15 or 16 or 17 or 18 or 19 35800

21 7 and 10 and 13 and 20 6

22 7 and 13 and 20 117
